# Supplementary material for: A likely role for stratification in long-term changes of the global ocean tides
Source: Commun Earth Environ. 2024 May 17;5(1):261. doi: 10.1038/s43247-024-01432-5 (PMC11549048; doi:10.1038/s43247-024-01432-5)
Supplement: Supplementary file 2 — Supplementary Information [file 43247_2024_1432_MOESM2_ESM.pdf]

A likely role for stratification in long-term changes of the global ocean tides: Supplementary Information

Lana Opel[1,*], **Michael Schindelegger**[1], **and Richard D. Ray**[2]

[1]Institute of Geodesy and Geoinformation, University of Bonn, Bonn, Germany
[2]Geodesy & Geophysics Laboratory, NASA Goddard Space Flight Center, Greenbelt, MD, USA
[*]opel@igg.uni-bonn.de

Supplementary Note 1

Validation

Supplementary Table 1 illustrates the accuracy of our M_2, S_2, K_1, and O_1 barotropic surface tide solutions in terms of a spatially averaged root mean square (RMS) error ($\overline{\Delta\eta}$) and the percentage of variance explained (PVE) relative to TPXO9 (updated version of ref.[1]). The corresponding mathematical expressions are[2,3]

$$\overline{\Delta\eta} = \left[\frac{\int\int |\hat{\eta} - \hat{\eta}_R|^2 \mathrm{d}A}{2\int\int \mathrm{d}A} \right]^{1/2} \quad (1)$$

$$\mathrm{PVE} = 100 \cdot \left[1 - \left(\frac{\overline{\Delta\eta}}{S} \right)^2 \right] \quad (2)$$

where $\hat{\eta}$ denotes the simulated tide (in complex notation), $\hat{\eta}_R$ is the reference tide from TPXO9, dA represents the surface element of the considered ocean domain, and the signal S is defined as

$$S = \left[\frac{\int\int \eta^2 \mathrm{d}A}{2\int\int \mathrm{d}A} \right]^{1/2} . \quad (3)$$

As is standard, we evaluate these metrics in latitudes equatorward of 66°, both for deep ($>$ 1,000 m) and shallow ($<$ 1,000 m) regions. In a similar vein, comparisons with “ground truth" M_2, S_2, K_1, and O_1 determinations are split into a deep-water and a coastal component. The respective test data are from 151 deep-ocean bottom pressure recorders[4] and the tide gauge network used in the main article. We list the RMS misfit with the in situ tidal estimates in Supplementary Table 1, calculated as in Eq. (1) without area weighting.

Supplementary Table 1. Validation of modeled barotropic and baroclinic surface tides

	M_2	S_2	K_1	O_1
Comparison with TPXO9[a], RMS misfit $\overline{\Delta\eta}$ (cm) and PVE (%)				
$>$ 1000 m	4.9 (96.6)	3.2 (90.8)	1.9 (96.1)	1.7 (93.0)
$<$ 1000 m	23.0 (78.5)	10.2 (69.9)	8.8 (78.0)	6.6 (76.4)
Comparison with in-situ data, RMS misfit $\overline{\Delta\eta}$ (cm)				
Deep-ocean seafloor gauges[b]	5.4	3.7	1.5	1.8
Coastal tide gauges, this study	32.5	19.2	4.9	3.9
Area-averaged amplitudes (cm) of internal tides[c]				
North Pacific	1.02 (0.98)	0.45 (0.38)	–	–
South Pacific	0.90 (0.85)	0.29 (0.19)	–	–
Madagascar	1.02 (0.76)	0.53 (0.30)	–	–
Philippines	–	–	0.79 (0.56)	0.60 (0.49)
Central Indian Ocean	–	–	0.38 (0.23)	0.24 (0.11)

[a]Statistics are for the spatially smoothed surface tide solutions of the year 2006 in latitudes lower than 66°; PVE values are given in parentheses.
[b]151 deep-ocean seafloor gauges are from ref.[4].
[c]Stationary baroclinic tidal signals are compared to the altimetry-based estimates of ref.[5] (in parentheses) over five rectangular domains, as marked out in Supplementary Fig. 1

Supplementary Figures

Supplementary Figs. 1–11 are shown in the order of their mentioning in the main article.

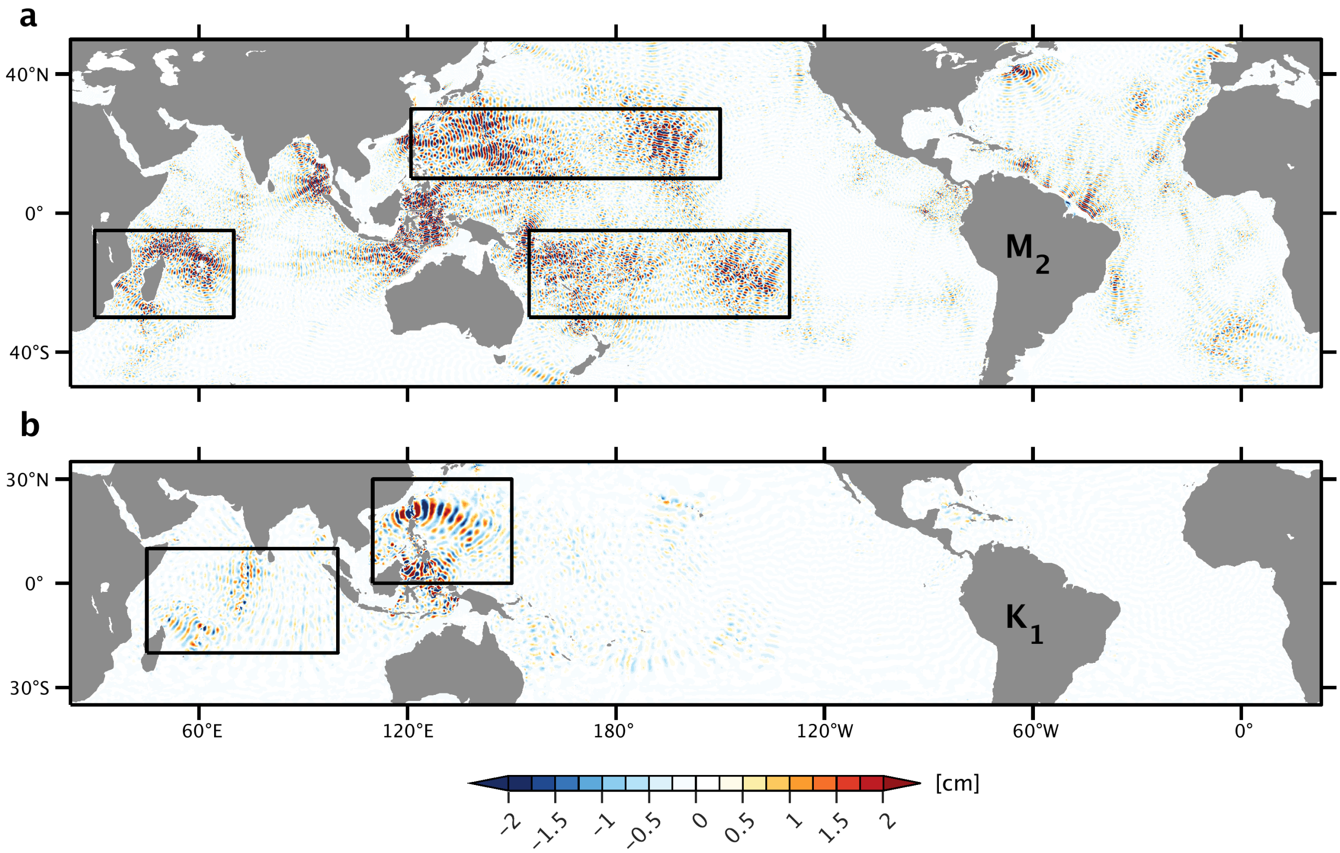

Supplementary Figure 1. In-phase component of the stationary **a** M_2 and **b** K_1 internal tide in surface elevation from one simulation (year 2006). Black boxes denote the regions used to compute the area-averaged internal tide amplitudes in Supplementary Table 1.

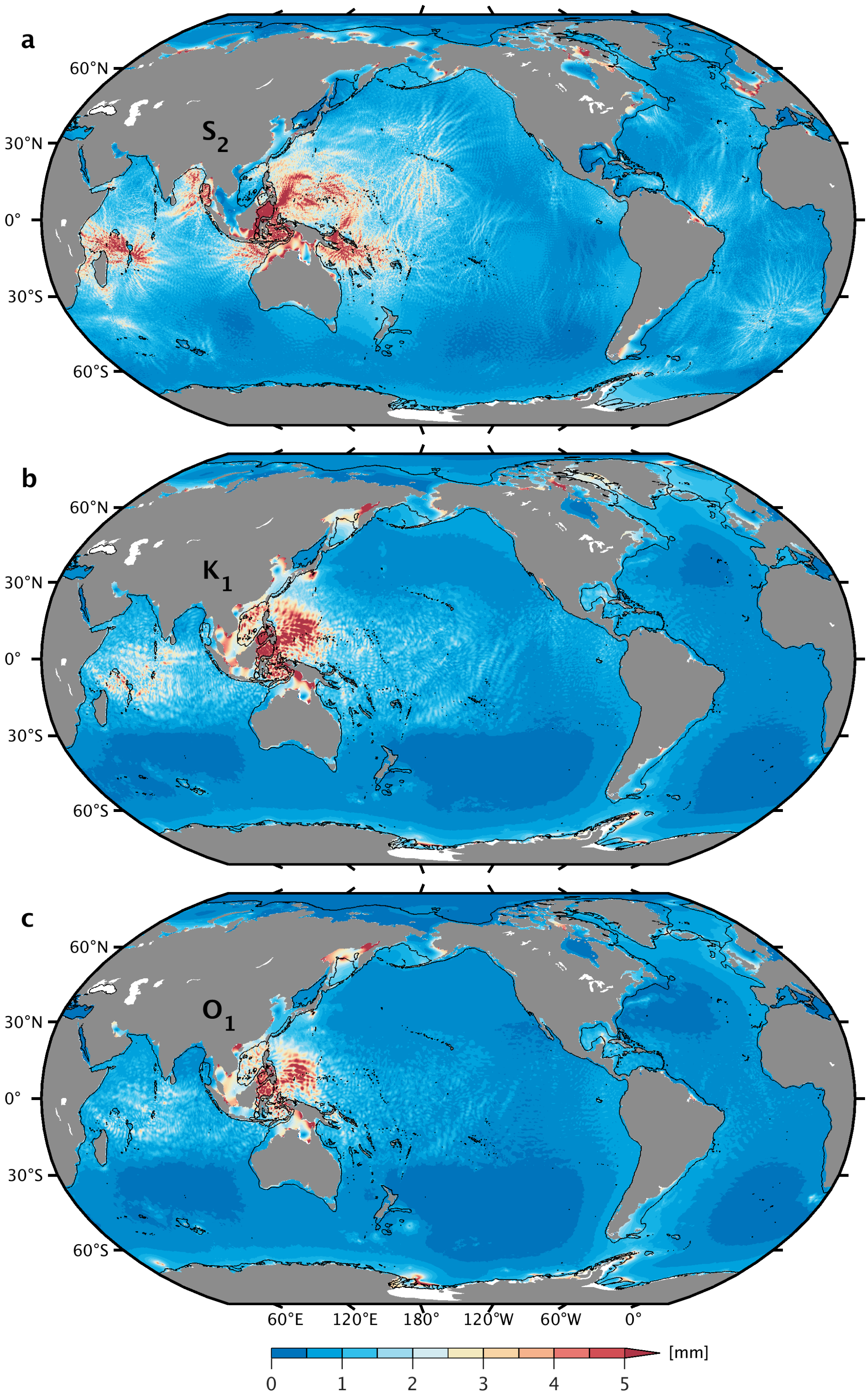

Supplementary Figure 2. Total RMS variability[6] (mm) of the **a** S_2, **b** K_1, and **c** O_1 surface tide in the simulations with stratification changes. Values are computed from annual tidal solutions, 1993–2020, without removing trends. The black solid line represents the 500 m isobath.

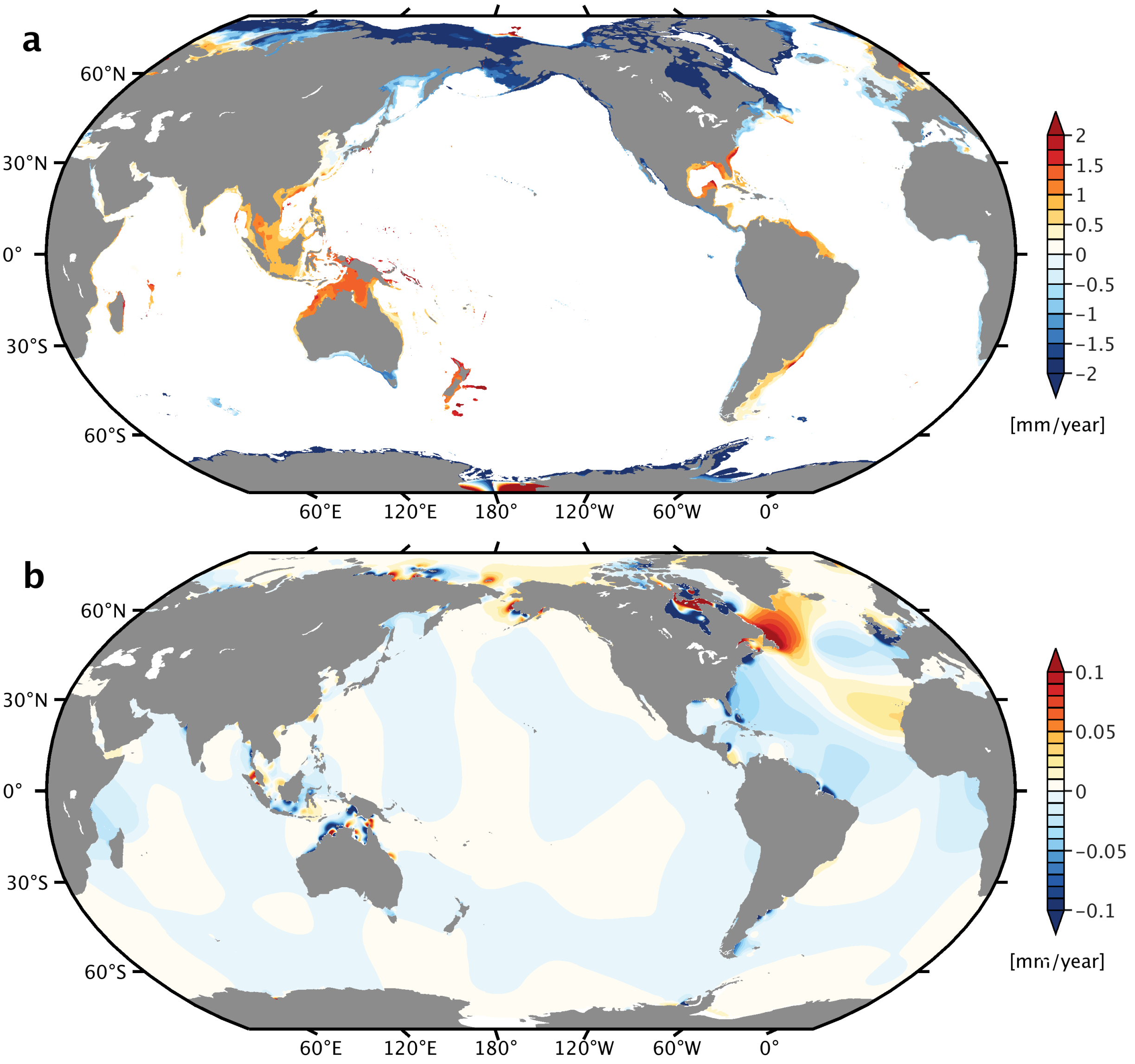

Supplementary Figure 3. a Trend in the MITgcm's mean free surface height across the 28 simulations and **b** the corresponding M_2 amplitude response (in mm yr^{-1}), as deduced from two endpoint simulations with perturbed and unperturbed water depths, see "Effects of sea level change" (Methods). Values at deep ocean grid points (> 500 m) are clipped in **a** to emphasize shallow regions were the tide is sensitive to water depth changes. Note that the trend signal in **b** was removed from all baroclinic modeling results for M_2 shown in the main paper.

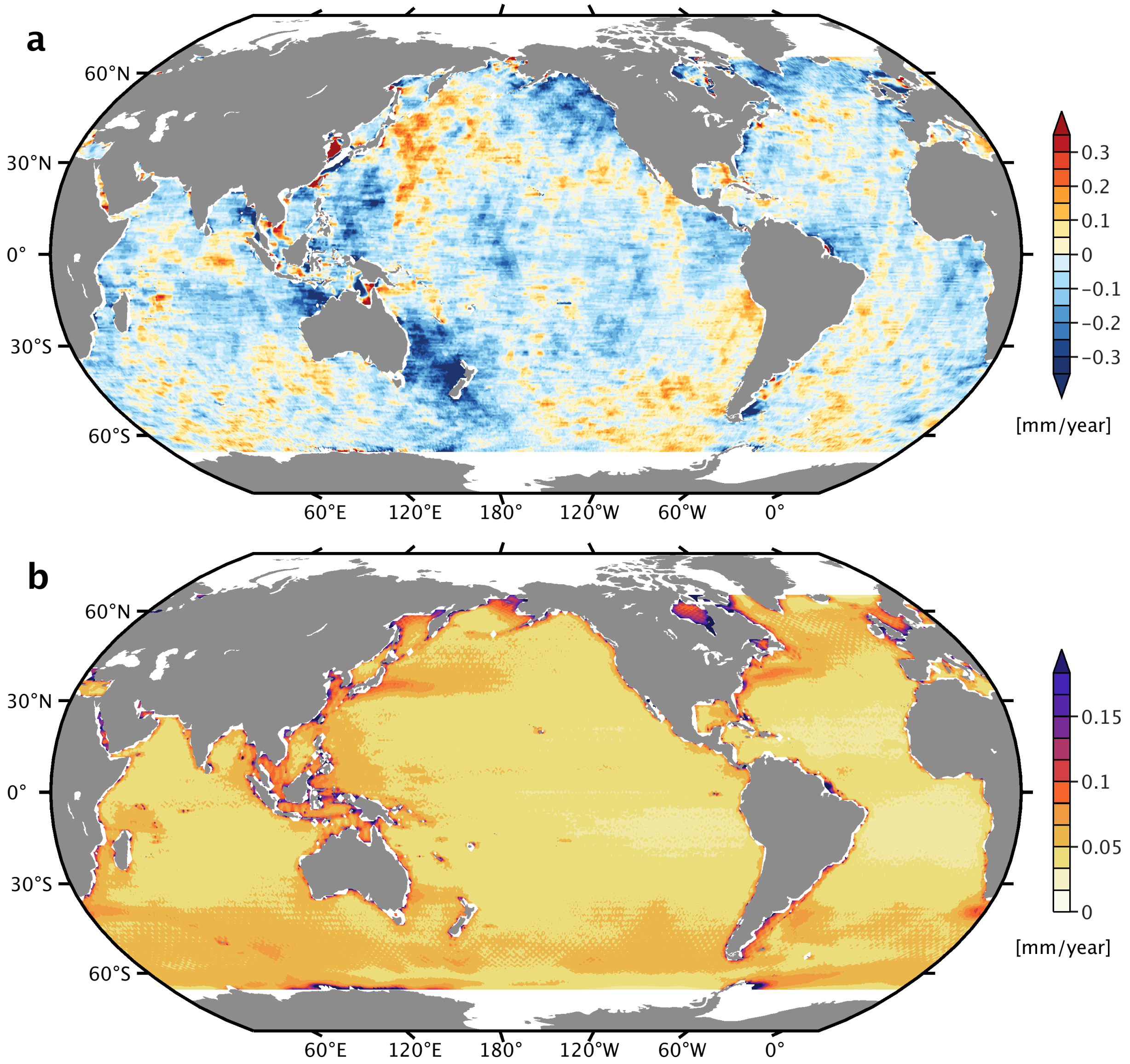

Supplementary Figure 4. Original (i.e., unsmoothed) altimetry-based solution for M_2 amplitude trends, 1993–2020. Shown are **a** trend values in mm yr^{-1} and **b** the associated onefold standard error.

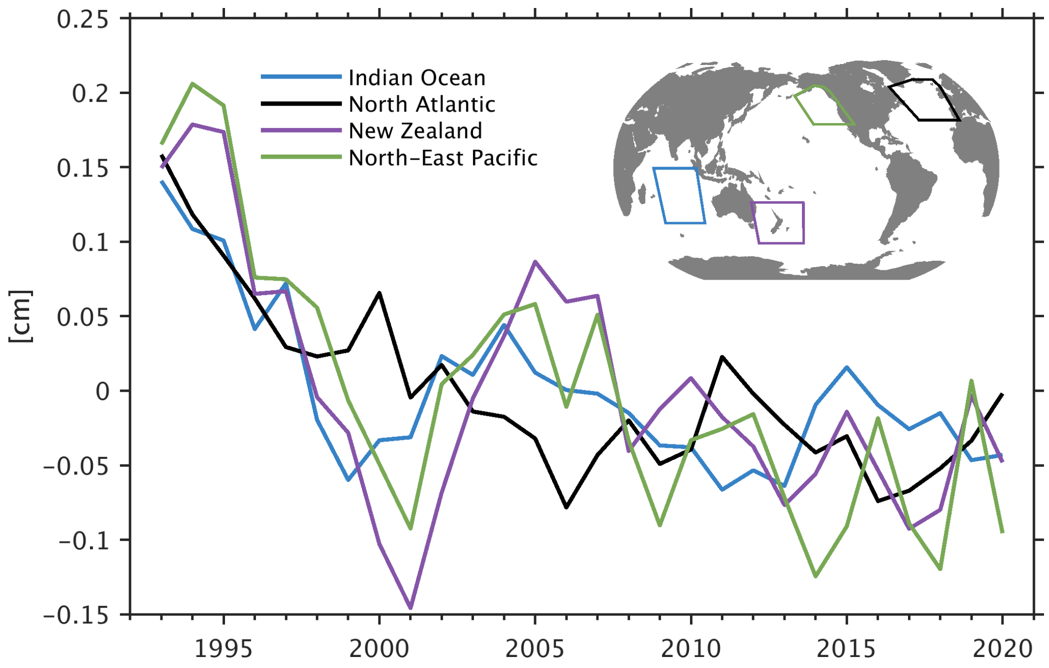

Supplementary Figure 5. Area-averaged annual barotropic M_2 amplitude anomalies (cm) from MITgcm simulations in four regions (shown as polygons on the map, top right corner). Corresponding trend estimates are given in Table 1 in the main text. Note that at any location, the altimetry solution (e.g., Supplementary Fig. 4) is a direct trend estimate and not a yearly-sampled time series.

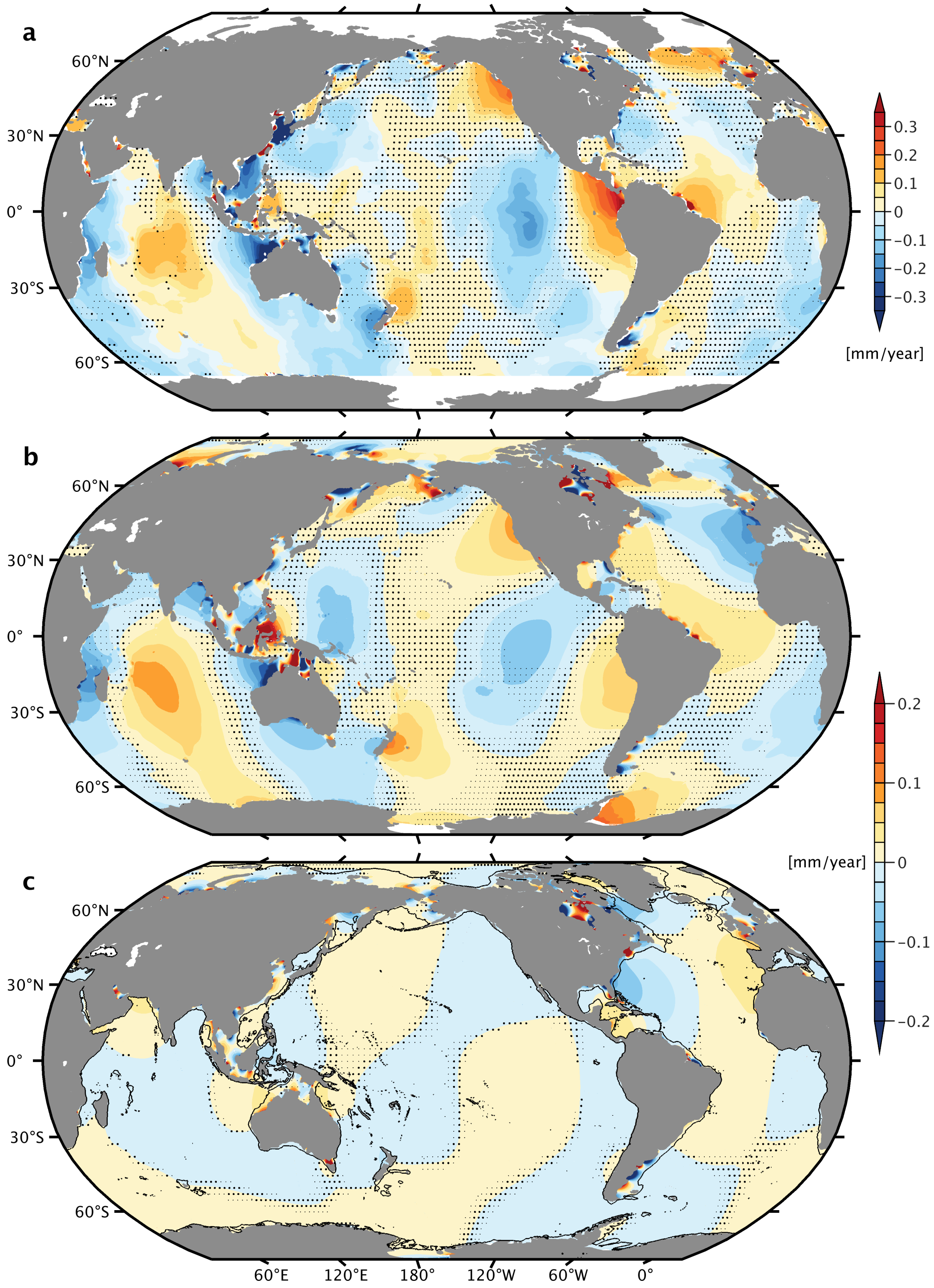

Supplementary Figure 6. Observed and modeled M_2 trends (1993–2020) as in Fig. 3 of the main text but for the in-phase component. Shown are **a** smoothed trends from satellite altimetry, along with simulated M_2 trends due to **b** stratification changes and **c** relative sea level rise. Note that the color axis in **a** extends to ± 0.35 mm yr^{-1}, a factor of 1.75 higher than in **b** and **c**. Heavy (or light) black dots identify regions where values do not pass the 68% (or 95%) threshold for statistical significance.

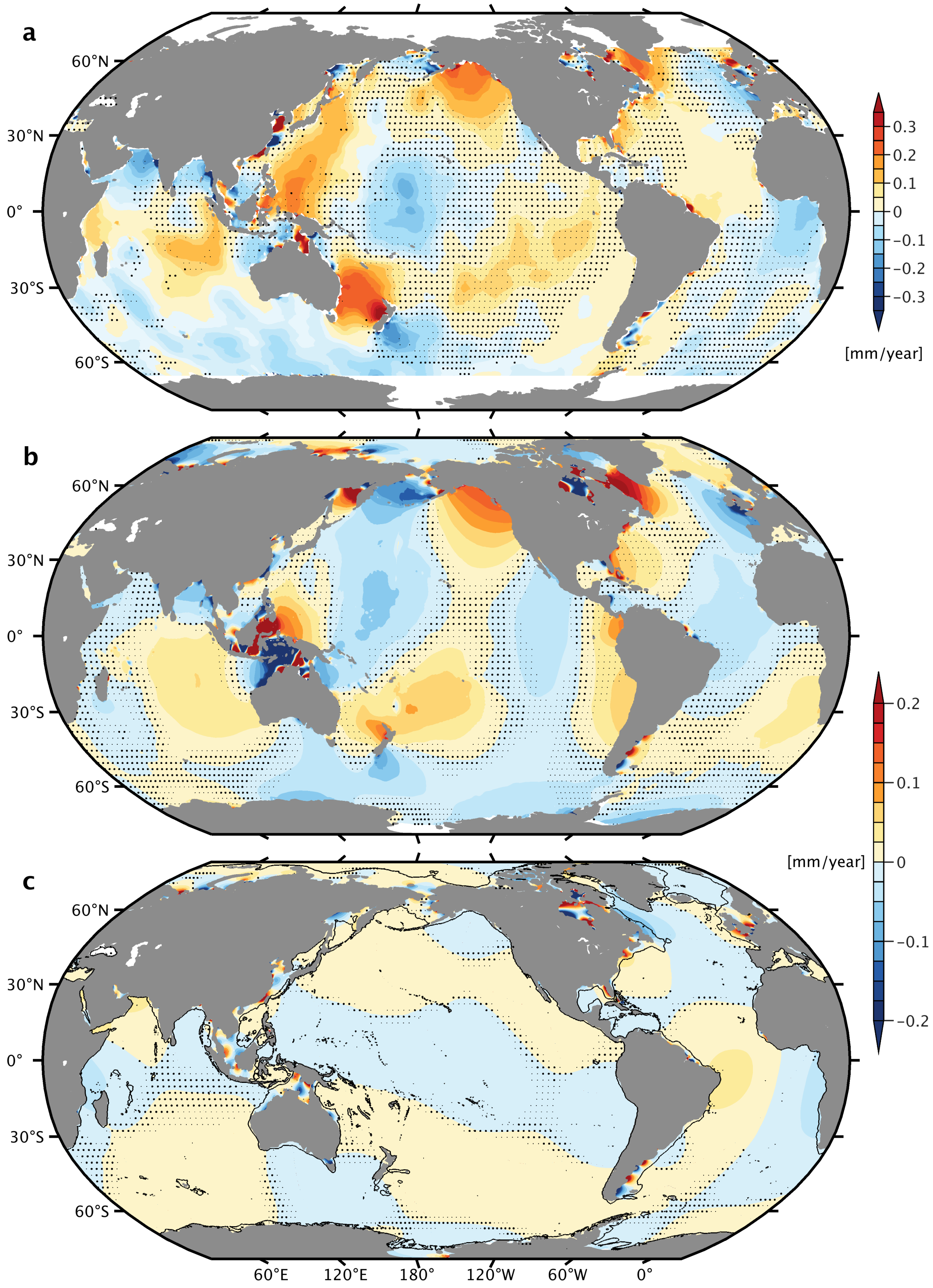

Supplementary Figure 7. As in Supplementary Fig. 6 but for the quadrature component of M_2.

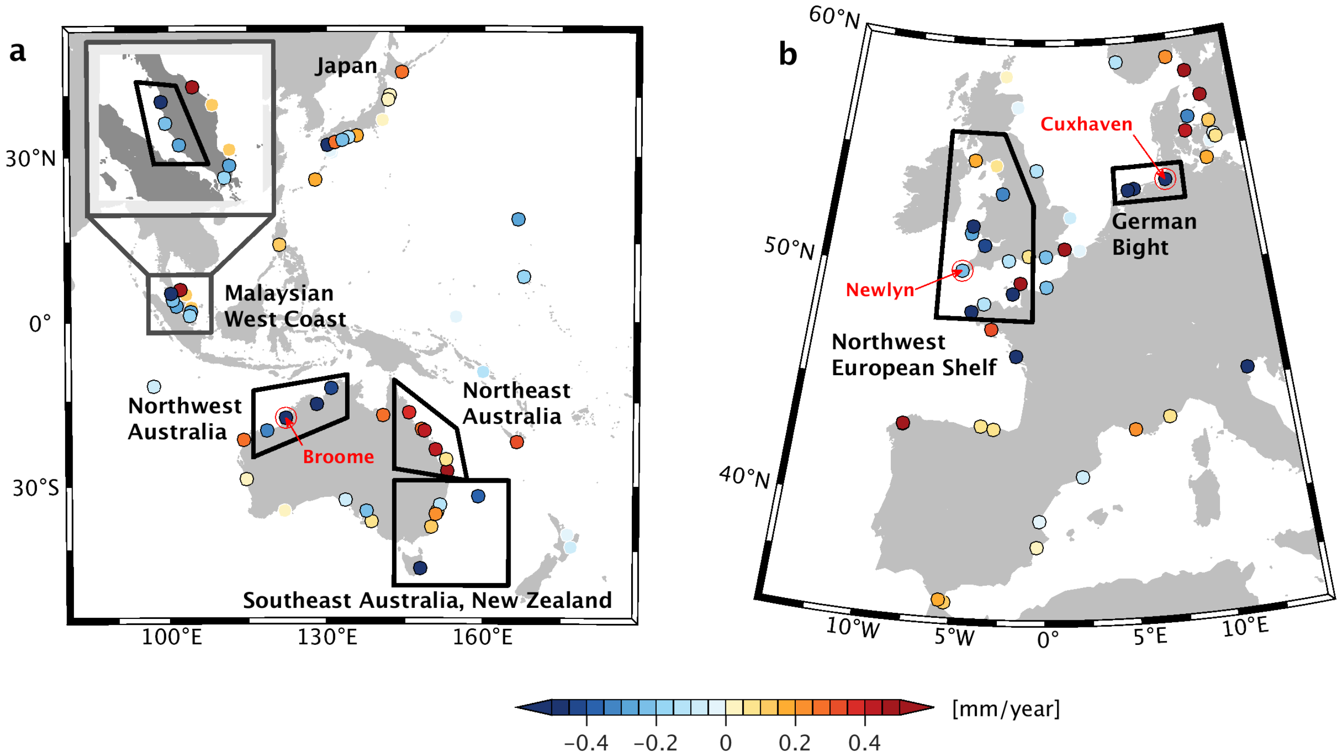

Supplementary Figure 8. Tide gauge estimates of M_2 amplitude trends (mm yr^{-1}) around Australia/Southeast Asia and Europe. Markers are highlighted with black (or respectively white) edges wherever fitted rates are statistically significant (insignificant) at the 68% confidence level. Polygons with black outline indicate the averaging regions underlying Fig. 7 in the main text.

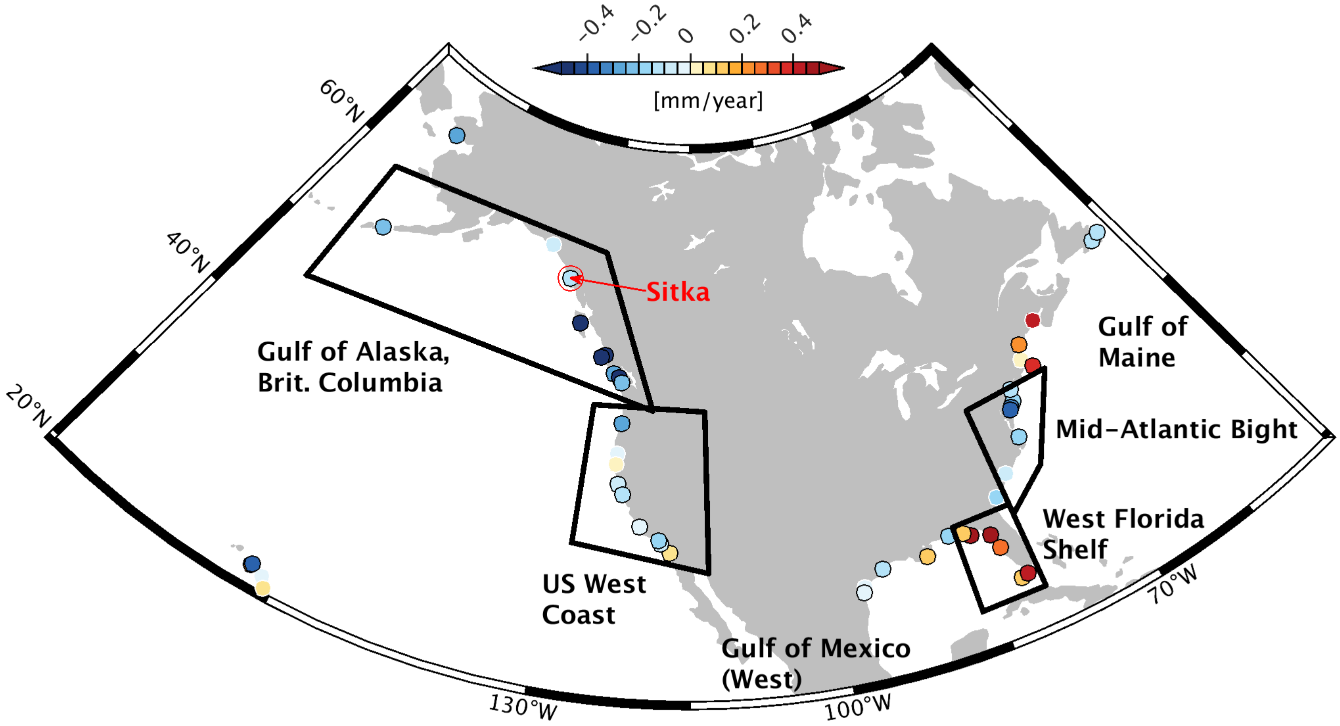

Supplementary Figure 9. As in Supplementary Fig. 8, but for the ocean and marginal seas encasing North America.

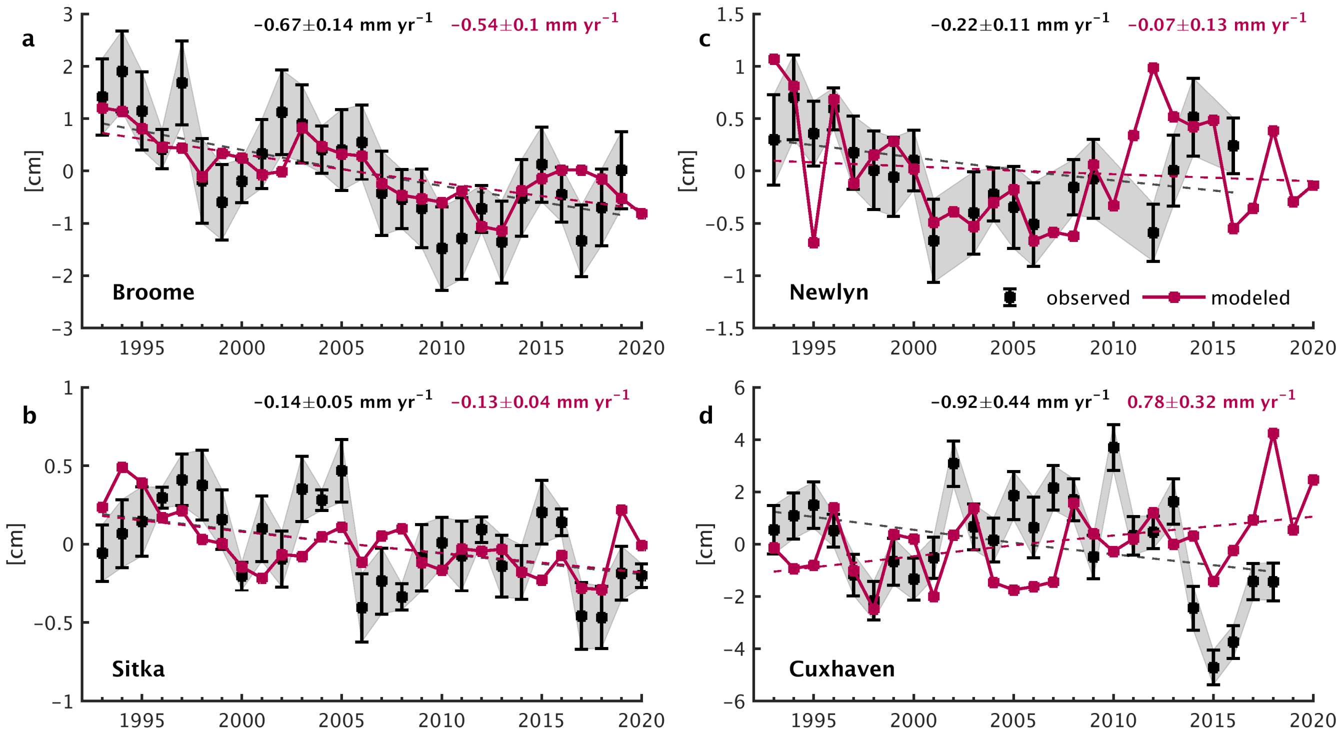

Supplementary Figure 10. Annual M_2 amplitude changes (cm), 1993–2020, at tide gauges **a** Broome, Australia, **b** Sitka, Canada, **c** Newlyn, United Kingdom, and **d** Cuxhaven, Germany, from observations (black markers, with standard errors) and simulations (magenta markers). Respective trend estimates and 68% confidence intervals are included in the top right corner. The simulation results represent the sum of sea level rise and stratification effects on the barotropic M_2 tide. Tide gauge locations are highlighted on Supplementary Figs. 8 and 9.

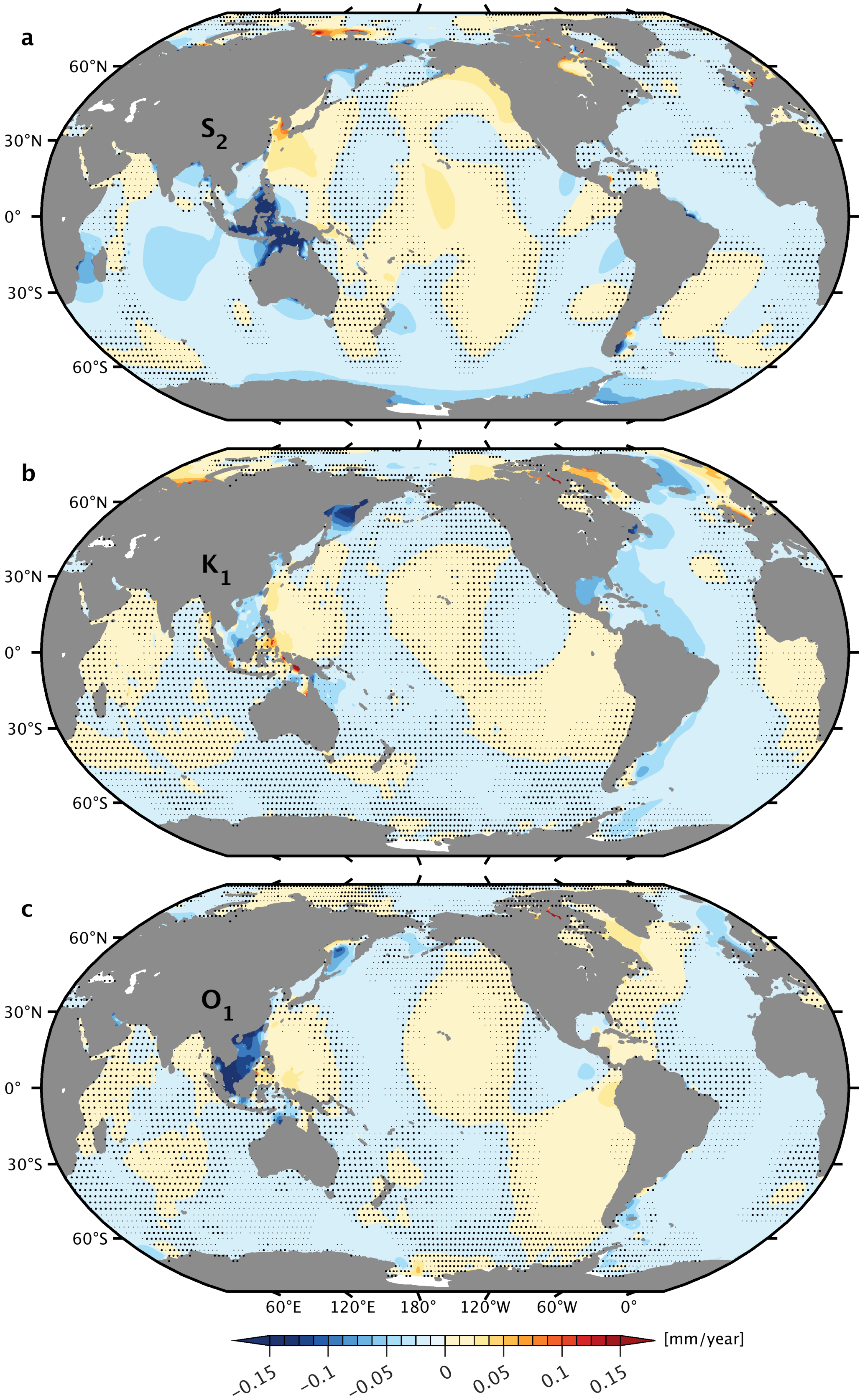

Supplementary Figure 11. Modeled trends (in mm yr^{-1}) of the **a** S_2, **b** K_1 and **c** O_1 barotropic tidal amplitudes from MITgcm simulations with annually varying stratification (1993–2020). Heavy (light) black dots identify regions where values do not pass the 68% (95%) level for statistical significance.

Supplementary Table 2. Secular trends in M_2 amplitude H at GESLA-3 tide gauges considered for spatial averaging; see Fig. 7 in the main text. 68% confidence intervals, time span, and number of full calendar years are also given.

GESLA-3 name	$\dot{H}$ (mm yr^{-1})		Time span	No. years
Gulf of Alaska, British Columbia				
dutch_harbor_ak-041b-usa-uhslc	−0.23	±0.04	1993–2018	26
unalaska-9462620-usa-noaa	−0.21	±0.04	1993–2020	25
yakutat-9453220-usa-noaa	−0.06	±0.06	1994–2019	24
sitka-9451600-usa-noaa	−0.14	±0.05	1993–2020	28
queen_charlotte_city-9850-can-meds	−1.20	±0.15	1997–2020	24
port_hardy_bc-8408-can-meds	−0.60	±0.09	1994–2020	25
winter_harbour_bc-8735-can-meds	−0.45	±0.08	1998–2020	23
tofino_bc-8615-can-meds	−0.27	±0.04	1997–2020	24
bamfield_bc-8545-can-meds	−0.48	±0.07	1993–2020	28
neah_bay-9443090-usa-noaa	−0.24	±0.06	1993–2020	28
US West Coast				
arena_cove-9416841-usa-noaa	−0.08	±0.04	1993–2020	28
crescent_city-9419750-usa-noaa	−0.04	±0.04	1993–2020	26
humboldt_bay_ca-576a-usa-uhslc	0.00	±0.05	1993–2018	24
la_jolla-9410230-usa-noaa	0.08	±0.05	1993–2020	27
los_angeles-9410660-usa-noaa	−0.08	±0.04	1993–2020	28
north_spit-9418767-usa-noaa	0.03	±0.04	1993–2020	27
point_reyes-9415020-usa-noaa	−0.13	±0.03	1993–2020	27
port_san_luis-9412110-usa-noaa	−0.03	±0.03	1993–2020	27
santa_monica-9410840-usa-noaa	−0.15	±0.04	1995–2020	25
south_beach-9435380-usa-noaa	−0.27	±0.04	1993–2020	28
West Florida Shelf				
apalachicola-8728690-usa-noaa	0.59	±0.09	1993–2020	26
cedar_key-8727520-usa-noaa	0.61	±0.27	1993–2020	21
key_west-8724580-usa-noaa	0.10	±0.02	1993–2020	27
panama_city-8729108-usa-noaa	0.13	±0.02	1993–2019	22
st_petersburg-8726520-usa-noaa	0.29	±0.10	1993–2019	27
vaca_key-8723970-usa-noaa	0.45	±0.12	1994–2020	24
Mid-Atlantic Bight				
atlantic_city-8534720-usa-noaa	−0.16	±0.07	1993–2020	25
cape_may-8536110-usa-noaa	−0.33	±0.12	1993–2019	25
duck_pier_nc-260a-usa-uhslc	−0.18	±0.05	1993–2018	23
lewes-8557380-usa-noaa	−0.39	±0.06	1993–2020	28
sandy_hook-8531680-usa-noaa	−0.14	±0.07	1993–2020	27
springmaid_pier-8661070-usa-noaa	−0.07	±0.07	1993–2020	24
Northwest European Shelf				
cherbourg_60minute-che-fra-cmems	0.45	±0.13	1993–2019	26
leconquet_60minute-lec-fra-cmems	−0.53	±0.16	1993–2019	22
newlyn_cornwall-294a-gbr-uhslc	−0.22	±0.11	1993–2016	19

portsmouth-ptm-gbr-bodc	0.09	±0.08	1993–2020	22
roscoff_60minute-ros-fra-cmems	−0.11	±0.11	1993–2019	23
st_helier_jersey-jer-gbr-bodc	−0.91	±0.21	1993–2015	22
sthelier-sth-gbr-cmems	−0.59	±0.16	1993–2020	25
weymouth-wey-gbr-cmems	−0.13	±0.06	1993–2020	21
workington-wor-gbr-cmems	0.07	±0.14	1993–2020	21
ilfracombe-ilf-gbr-cmems	−0.41	±0.22	1993–2018	18
milford-mil-gbr-cmems	−0.27	±0.15	1993–2019	15
fishguard-fis-gbr-cmems	−0.97	±0.07	1993–2019	19
liverpool-liv-gbr-cmems	−0.31	±0.12	1993–2020	15
portpatrick-por-gbr-cmems	0.20	±0.06	1993–2020	20
German Bight				
cuxhaven-825a-deu-uhslc	−0.92	±0.43	1993–2018	26
huibertgat-huibgt-nld-rws	−0.82	±0.15	1993–2017	24
wierumergronden-wiermgdn-nld-rws	−0.66	±0.17	1993–2017	25
Northwest Australia				
port_hedland-169a-aus-uhslc	−0.31	±0.09	1993–2018	25
broome-62650-aus-bom	−0.67	±0.14	1993–2019	27
wyndham-165a-aus-uhslc	−2.03	±0.59	1994–2018	23
darwin-168a-aus-uhslc	−0.42	±0.18	1993–2018	25
Northeast Australia				
bowen-59320-aus-bom	0.28	±0.09	1993–2019	25
brisbane_bar-59980-aus-bom	0.66	±0.11	1993–2019	26
bundaberg-332a-aus-uhslc	−0.04	±0.08	1993–2018	26
cairns-59060-aus-bom	0.36	±0.15	1993–2019	26
port_alma-59690-aus-bom	0.44	±0.11	1993–2019	25
shute_harbour-59410-aus-bom	0.44	±0.11	1994–2017	22
urangan-59850-aus-bom	0.08	±0.08	1995–2019	21
Southeast Australia, New Zealand				
bermagui-219470-aus-bom	0.11	±0.05	1993–2019	23
botany_bay-60390-aus-bom	0.07	±0.07	1993–2019	23
lord_howe_island-57720-aus-bom	−0.39	±0.07	1995–2019	21
napier-668a-nzl-uhslc	−0.07	±0.09	1993–2018	21
newcastle-60310-aus-bom	−0.14	±0.08	1993–2019	27
port_kembla-60420-aus-bom	0.23	±0.07	1993–2019	27
spring_bay-61170-aus-bom	−0.64	±0.09	1993–2019	27
tauranga-073a-nzl-uhslc	−0.05	±0.16	1993–2018	21
Malaysian West Coast				
kelang-140a-mys-uhslc	−0.27	±0.26	1993–2012	16
lumut-143a-mys-uhslc	−0.21	±0.10	1995–2014	15
penang-144a-mys-uhslc	−0.69	±0.19	1994–2014	19

Supplementary References

1. Egbert, G. D. & Erofeeva, S. Y. Efficient inverse modeling of barotropic ocean tides. *J. Atmospheric Ocean. Technol.* **19**, 183–204, DOI: https://doi.org/10.1175/1520-0426(2002)019<0183:EIMOBO>2.0.CO;2 (2002).
2. Arbic, B. K., Garner, S. T., Hallberg, R. W. & Simmons, H. L. The accuracy of surface elevations in forward global barotropic and baroclinic tide models. *Deep. Res. II* **51**, 3069–3101, DOI: 10.1016/j.dsr2.2004.09.014 (2004).
3. Stammer, D. *et al.* Accuracy assessment of global barotropic ocean tide models. *Rev. Geophys.* **52**, 243–282, DOI: https://doi.org/10.1002/2014RG000450 (2014).
4. Ray, R. D. Precise comparisons of bottom-pressure and altimetric ocean tides. *J. Geophys. Res. Ocean.* **118**, 4570–4584, DOI: https://doi.org/10.1002/jgrc.20336 (2013).
5. Zaron, E. D. Baroclinic tidal sea level from exact-repeat mission altimetry. *J. Phys. Ocean.* **49**, 193–210, DOI: https://doi.org/10.1175/JPO-D-18-0127.1 (2019).
6. Shriver, J. F., Richman, J. G. & Arbic, B. K. How stationary are the internal tides in a high-resolution global ocean circulation model? *J. Geophys. Res. Ocean.* **119**, 2769–2787, DOI: 10.1002/2013JC009423 (2014).
